# Clinical examination of the knee: know your tools for diagnosis of knee injuries

**DOI:** 10.1186/1758-2555-3-25

**Published:** 2011-10-28

**Authors:** Roberto Rossi, Federico Dettoni, Matteo Bruzzone, Umberto Cottino, Davide G D'Elicio, Davide E Bonasia

**Affiliations:** 1 SCDU Ortopedia e Traumatologia, Ospedale Mauriziano Umberto I, Largo Turati 62, Torino, 10128, Italy; 2 Universita' degli Studi di Torino, via Verdi 8, Torino, 10124, Italy; 3 I Clinica Ortopedia e Traumatologia, Ospedale CTO, via Zuretti 29, Torino, 10126, Italy

## Abstract

The clinical evaluation of the knee is a fundamental tool to correctly address diagnosis and treatment, and should never be replaced by the findings retrieved by the imaging studies carried on the patient.

Every surgeon has his own series of exams with whom he is more confident and on whom he relies on for diagnosis. Usually, three sets of series are used: one for patello-femoral/extensor mechanism pathologies; one for meniscal and chondral (articular) lesions; and one for instability evaluation.

This review analyses the most commonly used tests and signs for knee examination, outlining the correct way to perform the test, the correct interpretation of a positive test and the best management for evaluating an injured knee both in the acute and delayed timing.

## Introduction

The introduction of highly effective imaging tools like Computed Tomography and Magnetic Resonance in the clinical practice in Orthopaedics and Traumatology has stolen the central role of clinical evaluation, so that nowadays there's a common feeling, between patients but also between surgeons, that the diagnosis of a thorn meniscus or a ruptured ACL has to be ruled out only on the basis of an imaging study.

But the efficacy and affordability of a correct clinical examination needs not to be forgotten: this paper presents an overview of the most known tests and signs for knee examination, grouped in the three aspects of knee injuries: 1) patello-femoral joint/extensor mechanism; 2) articular (meniscal and chondral) lesions; and 3) knee instability.

## Patient Interview

In all cases, the clinical evaluation should be introduced by a careful interview of the patient, in order to address the subsequent exam to the affected area of the knee, and to choose the correct series of tests and signs.

The beginning of the interview should be carried in order to 1) localize the pain/dysfunction in one aspect of the knee (extensor mechanism; articular: medial vs lateral vs patellofemoral; ligaments on the medial vs lateral compartment vs central pivot); 2) define the timing of onset of the injury/dysfunction: acute vs previous injury; chronic disease; overuse; 3) collect the actual symptoms felt by the patient: pain vs discomfort vs disability.

Extensor mechanism pathology is often related to a chronic, repetitive trauma. Nevertheless, recent injuries should be enquired, as anterior knee pain can be associated to a recent patellar subluxation or dislocation, or to ruptured patellar or quadriceps tendons, particularly in older patients.

Anterior pain during activity and at rest is mostly associated with chondral lesions, while pain associated with prolonged flexion can be raised by a slight instability or malalignement.

Meniscal lesions are almost always consequence of a single trauma, but chronic lesions and degenerative tears of the menisci as well as chondral defects secondary to overuse should not be forgotten. The interview should be focused on the mechanism of injury (direct trauma, sprain, complex trauma) and on the pre-existing condition of the knee (e.g. previous injuries, history of overuse). Most patients do not report a real trauma, but rather an acute pain occurred after a weight-bearing twist on the knee or a knee flexion.

Locking of the knee is usually associated with bucket handle tears of the meniscus, and must be carefully inquired. An haemorrhage around the posterior capsule and medial collateral ligament, with subsequent hamstring spasm can mimic a locking. Snaps, clicks, catches or jerks can be reported by the patients and the examiner should try to reproduce them with the manipulative manoeuvres.

Painful giving away of the knee is a common symptom, and is often reported as caused associated to rotatory movements and often associated with a feeling of "the joint jumping out of place". This symptom is non-specific and also reported in case of loose bodies, patellar chondromalacia, instability, quadriceps weakness.

In case of instability, the onset of the lesion can most of the times be related to a single injury, and the patient usually remembers it. However, it is often difficult to recall the exact mechanism of injury, and the patient should be forced in trying to reproduce the "twist" or impact sustained by the knee at the time of the injury: this can strongly help in estimating the anatomic structure(s) involved in the lesion. Additionally, the rupture of ligament such as ACL and PCL produces many times an audible "snapping" or "cracking" sound: the patient should be questioned if he/she heard such a sound.

## Clinical Examination

The clinical examination of a knee is addressed to evaluate three aspects: 1) patello-femoral joint/extensor mechanism; 2) articular (meniscal and chondral) lesions; and 3) knee instability.

The series of the most known exams, signs and tests used for each of the three aspects will be here discussed.

### 1-Patello-Femoral Joint

#### Q Angle

The Q-angle is the intersection between a line drawn from the anterior superior iliac spine to the center of the patella and a line drawn from the center of the tibial tubercle to the center of the patella. The angle can be measured in full knee extension, but the patella had better be centered in the trochlear groove to be more stable; therefore it is recommend to measure the Q-angle at 30° of knee flexion to move the patella into the proximal portion of the trochlea. The range of normality is usually considered 10° to 20°. An increased Q-angle may indicate a tendency to lateral tilt or glide. However, the clinical usefulness of the Q-angle is debated, and it has been reported no correspondence between Q-angle and PF pain measurements with patients' clinical symptoms [1]. A sitting Q angle (tubercle sulcus angle) has been advocated to better represent the relationship between the patellar and quad tendon vectors. An increased Q-angle (15°-20°) is associated to lateral patellar subluxation [2]. It has been noted that a static measure should not be used to assess a dynamic condition such as PF maltracking. It is still debated whether Q-angle correlates to PF pain syndrome [3] or not [4], and as far as this topic is still unclear, the Q-angle alone should never be used as a diagnostic tool for PF joint pathology.

#### Patellar Tilt and Glide

Patellar tilt and glide are often cited together, and many times are considered synonyms. Actually the patellar tilt indicates tightness of lateral restraints; it is performed with the patient supine with the knee in full extension. If the lateral side of the patella can not be elevated above the horizontal the test is positive.

**Figure 1:**
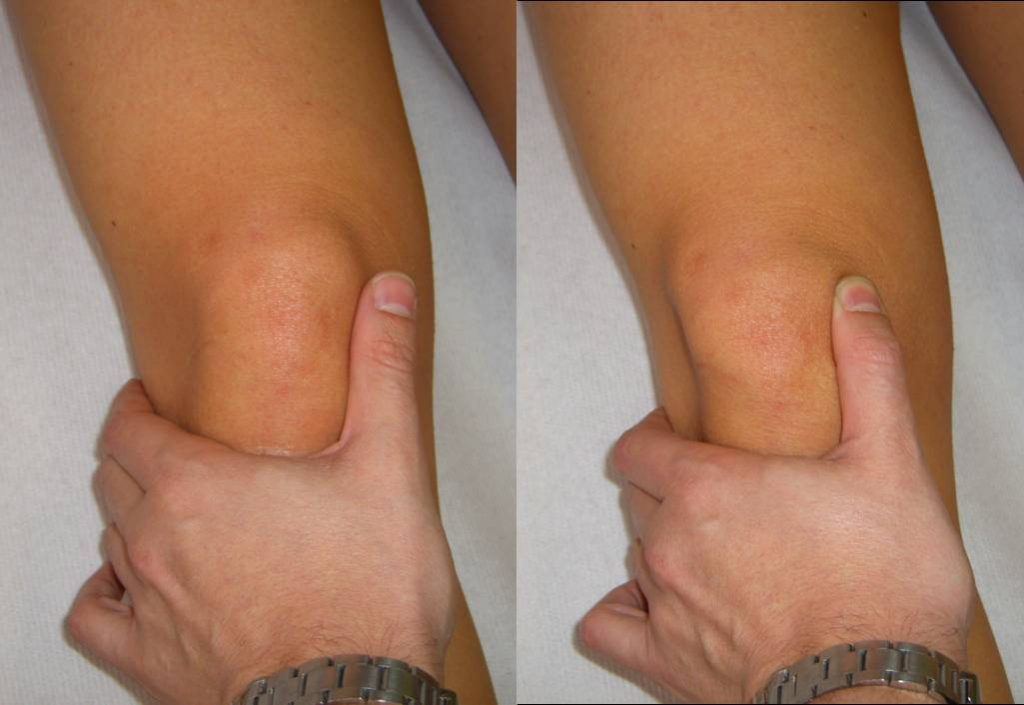
**Patellar Glide (Adapted with permission from: Rossi R, Bruzzone M, Dettoni F, Margheritini F: Clinical examination of the knee**. In: Orthopedic Sports Medicine, Principles and Practice. Edited by Margheritini F, Rossi R. Milan: Springer; 2010).

The glide test is performed with the knee flexed at 30°: if the patella glides laterally over 75% of its width, a medial restraints laxity is diagnosed; while when it glides less than 25%, lateral restraints tightness is predicted [5] (Figure 1).

**Figure 2:**
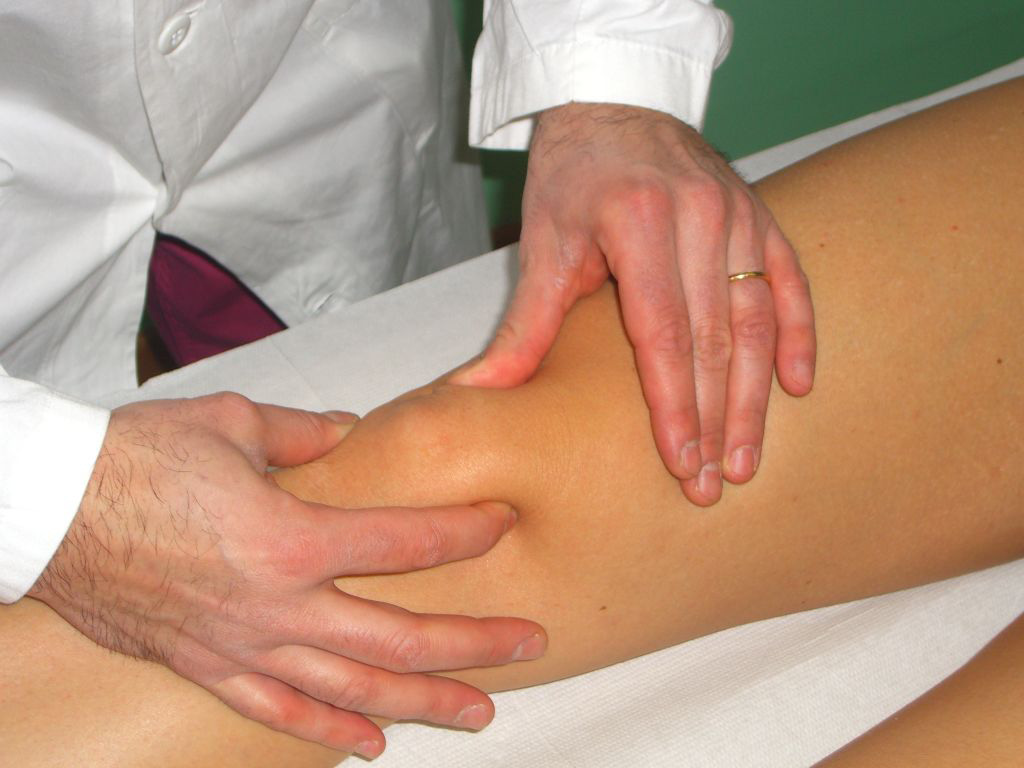
**MPFL palpation test (Adapted with permission from: Rossi R, Bruzzone M, Dettoni F, Margheritini F: Clinical examination of the knee**. In: Orthopedic Sports Medicine, Principles and Practice. Edited by Margheritini F, Rossi R. Milan: Springer; 2010).

The main restraint to the lateral dislocation is the medial patellar femoral ligament (MPFL). The MPFL can be evaluated with the knee in full extension and the patella medially subluxated with the thumb as in the glide test. This manoeuvre tightens the MPFL; if an area of tenderness is palpated, this usually identifies the location of the tear. A lateral glide greater than 75% of the patellar width is abnormal and indicates MPFL insufficiency (Figure 2).

#### Patella tracking

A careful observation of patella tracking is mandatory, to rule out any muscular/ligamentous deficiency.

The quadriceps muscle is composed by: the rectus femoris and vastus intermedius muscles, that apply an axial load to the patella; the vastus lateralis and vastus medialis which have oblique insertions pull the patella in either direction.

The medial retinaculum and the lateral retinaculum act as static constraint to patella tracking.

The tracking of the patella from full extension into flexion should be recorded visually, and should be smooth, without abrupt or sudden movements. During knee flexion the patella moves more centrally and the facets increase their contact with femoral condyles. The iliotibial band has an expansion to the lateral retinaculum, and during the knee flexion determines a lateralization of the patella. Lateralization of the patella during flexion can be determined by weakness of medial muscles and retinaculum, or tightness of lateral structures.

Additionally, the patella engages the femoral condyles (the trochlea) at about 20 to 30 degrees of knee flexion: in case of condyles hypoplasia the facets do not engage in the trochlea and the patella can glide easily.

Besides maltracking, any articular pain or crackling can be elicited by applying pressure on the patella during flexion and extension: this does not always indicate PF chondromalacia, but other causes of pain must be considered, such as neuroma, patellar tendonitis, plica, referred pain, meniscus derangement, synovitis, and osteocondritis dissecans [6].

#### J sign

When the patella is subluxated laterally and, it suddenly shifts medially, when engaging the femoral trochlea, following a J shaped path. This sign indicates excessive lateral patellar shift in terminal extension [7]; external rotation facilitate its identification [8].

### 2. Meniscal and chondral lesions

A meniscal tear can be difficult to diagnose, as symptoms are mostly non-specific and other injuries can disguise the meniscal lesion. A meniscal lesion has to be suspected every time in case of pain occurred after a weight-bearing sprain of the knee or after a prolonged squatting or a real trauma.

Chondral lesions are more often related to chronic degeneration, but acute lesions can also occur.

As the menisci have no direct innervation, pain is related to synovitis in the adjacent capsular and synovial tissues, as it happens for chondral lesions. Thus, discriminating between meniscal and chondral lesions can be sometimes difficult.

Crepitation during flexion and extension against resistance may indicate cartilage pathology. The patient may walk with an externally rotated gait to avoid contact of the medial femoral condyle with the medial tibial spine in case of chondral lesion at that level[9].

All tests for meniscal and chondral lesions are a combination of knee flexion, tibial rotation and a stress on the joint line: this is the position where the posterior condyles roll back and the joint space becomes narrow, tightly engaging the menisci.

The tests can be dived in palpation test (McMurray's, Bragard's, Steinmann's second, figure of four meniscal stress manoeuvre) and rotation test (Apley's, Bohler's, duck walking, Helfet's, Merke's, Payr's, Steimann's first) [10].

#### Meniscal Palpation Tests

**Figure 3:**
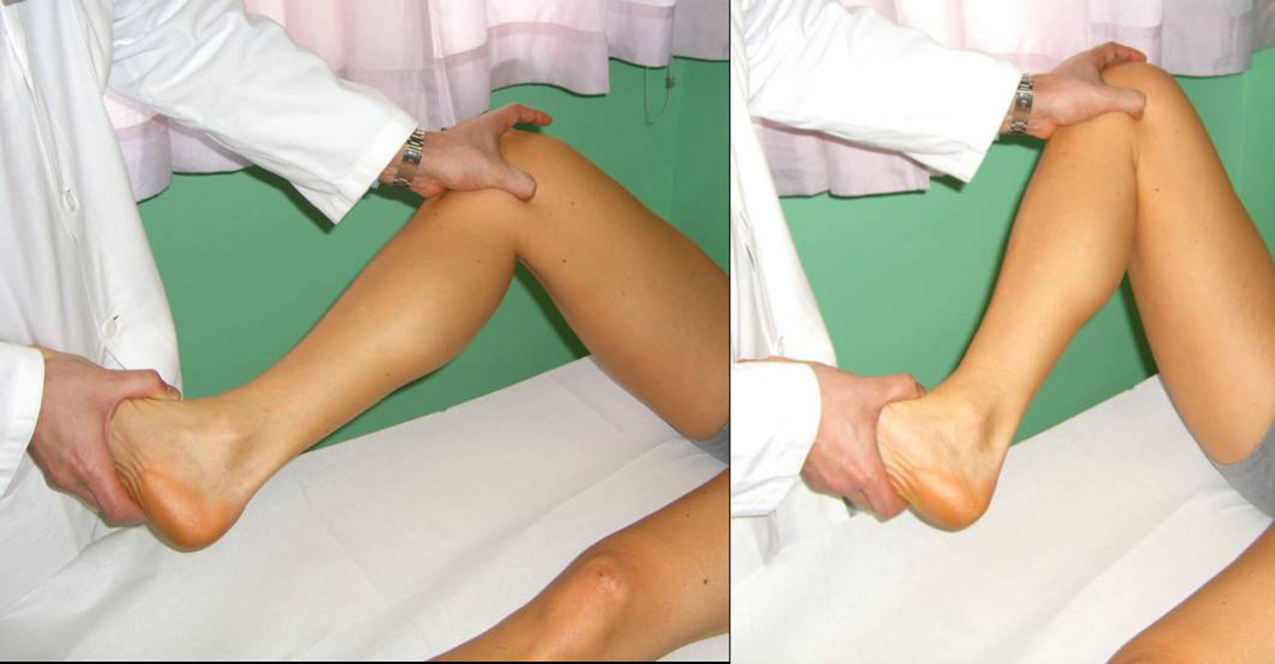
**McMurray Test (Adapted with permission from: Rossi R, Bruzzone M, Dettoni F, Margheritini F: Clinical examination of the knee**. In: Orthopedic Sports Medicine, Principles and Practice. Edited by Margheritini F, Rossi R. Milan: Springer; 2010).

In *McMurray test *the knee is flexed while the leg is externally rotated, palpating the joint line with a finger. Then, the knee is slowly extended. The test for lateral meniscus is carried out by internally rotating the leg. Pain or a crackling sound is felt when the condyle engages in the meniscal lesion (Figure 3).

In *Bragard's test*, external tibial rotation and knee extension bring the meniscus more anterior: if tenderness is felt along the joint line palpation, an articular surface irregularity (i.e. chondral lesion) or a meniscal tear is suspected.

In *Steinmann's second test *joint line tenderness migrates posteriorly with knee flexion and anteriorly with knee extension, following the movements of the meniscus.

**Figure 4:**
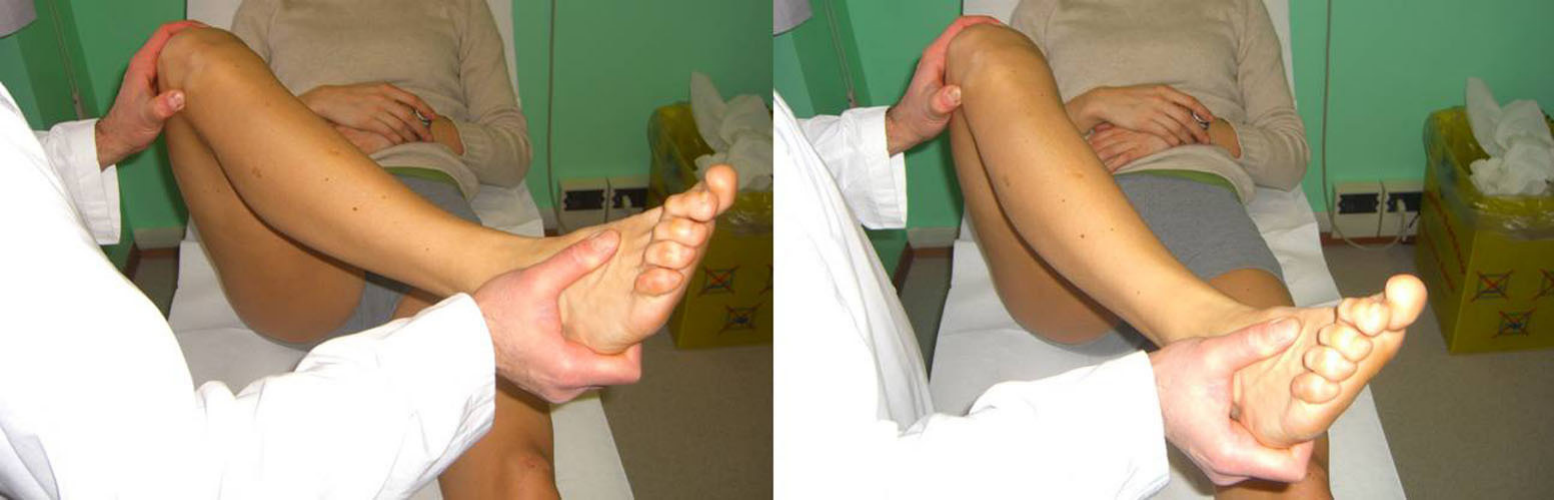
**The figure of four meniscal stress maneouver (Adapted with permission from: Rossi R, Bruzzone M, Dettoni F, Margheritini F: Clinical examination of the knee**. In: Orthopedic Sports Medicine, Principles and Practice. Edited by Margheritini F, Rossi R. Milan: Springer; 2010).

In the *figure of four meniscal stress manoeuvre*, the knee is held in a "figure of 4" (Cabot's) position, then the knee swings rapidly from a varus to a valgus stress, while a finger is pushed in the joint line. This brings the meniscus toward the periphery of the joint while the finger pushes it toward the centre of the joint: the combination of these two opposite forces stresses the meniscus, raising a sharp pain in case of meniscal tear (Figure 4).

#### Meniscal Rotation Tests

**Figure 5:**
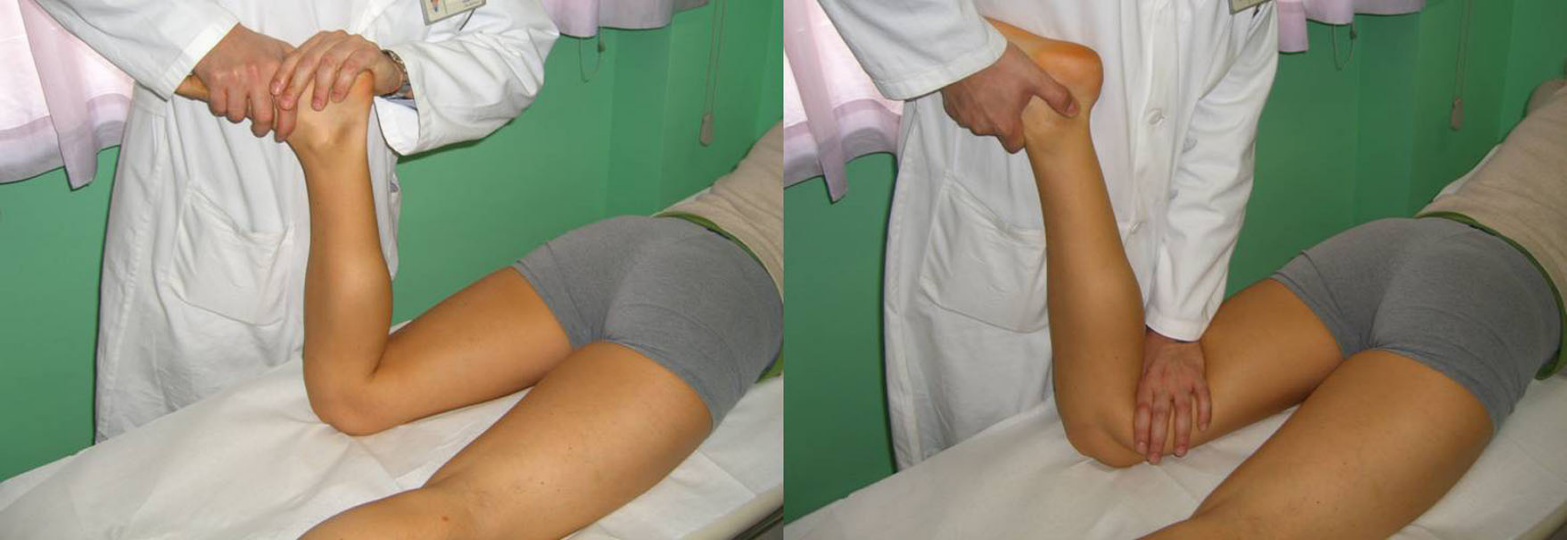
**Apley grinding test (Adapted with permission from: Rossi R, Bruzzone M, Dettoni F, Margheritini F: Clinical examination of the knee**. In: Orthopedic Sports Medicine, Principles and Practice. Edited by Margheritini F, Rossi R. Milan: Springer; 2010)

*Apley's (grinding) test *is carried out with the patient prone and the knee flexed to 90°. Then the leg is twisted and pulled, then pushed. If pain is felt only while pushing, a meniscal lesion is diagnosed, while if no difference between distraction and compression is detected, a chondral lesion is more likely (Figure 5).

In *Bohler's test *a varus stress and a valgus stress are applied to the knee: pain is elicited by compression of the meniscal tear.

*Squat test, duck walking test and Thessaly test *consist in several repetitions of full weightbearing flexions on the knee, in different positions (squatting, walking in full flexion, and at 5 and 20° flexion, respectively) [11].

*Merke's test *is similar to Thessaly test performed with the patient in a weightbearing position: pain with internal rotation of the body produces an external rotation of the tibia and medial joint line pain when medial meniscus is torn. The opposite occurs when lateral meniscus is torn.

In *Helfet's test *the knee is locked, and cannot rotate externally while extending, and the Q angle cannot reach normality with extension.

In *Peyr's test *the patient is asked to sit in Turkish position, thus stressing the medial joint line: if the position raises pain, the test is positive for a medial meniscal lesion.

In *Steinmann's first test *the knee is held flexed at 90° and forced to external rotation, then internal rotation: the test is positive for medial meniscal tear if raises pain upon externally rotating, while it is positive for lateral meniscal tears in case of pain during internal rotation.

### 3. Knee Instability

Instability is usually defined with a direction (anterior, posterior, medial, lateral, rotatory), which is the position the proximal tibia can abnormally reach, with respect to the distal femur. The direction of instability depends on the single, or multiple, structures involved: the main structures involved in knee (in)stability are: ACL, PCL, MCL, LCL, posterolateral corner and posteromedial corner.

Many manoeuvres are available to rule out the type of instability and test the knee structures involved. All tests can be divided in 4 groups: stress tests, slide tests, pivot shift (jerk) tests and rotational tests [6,9,10,12].

#### Stress Tests

The standard stress tests include valgus (abduction) and varus (adduction) tests; additionally, Cabot manoeuvre is a commonly used stress test.

**Figure 6:**
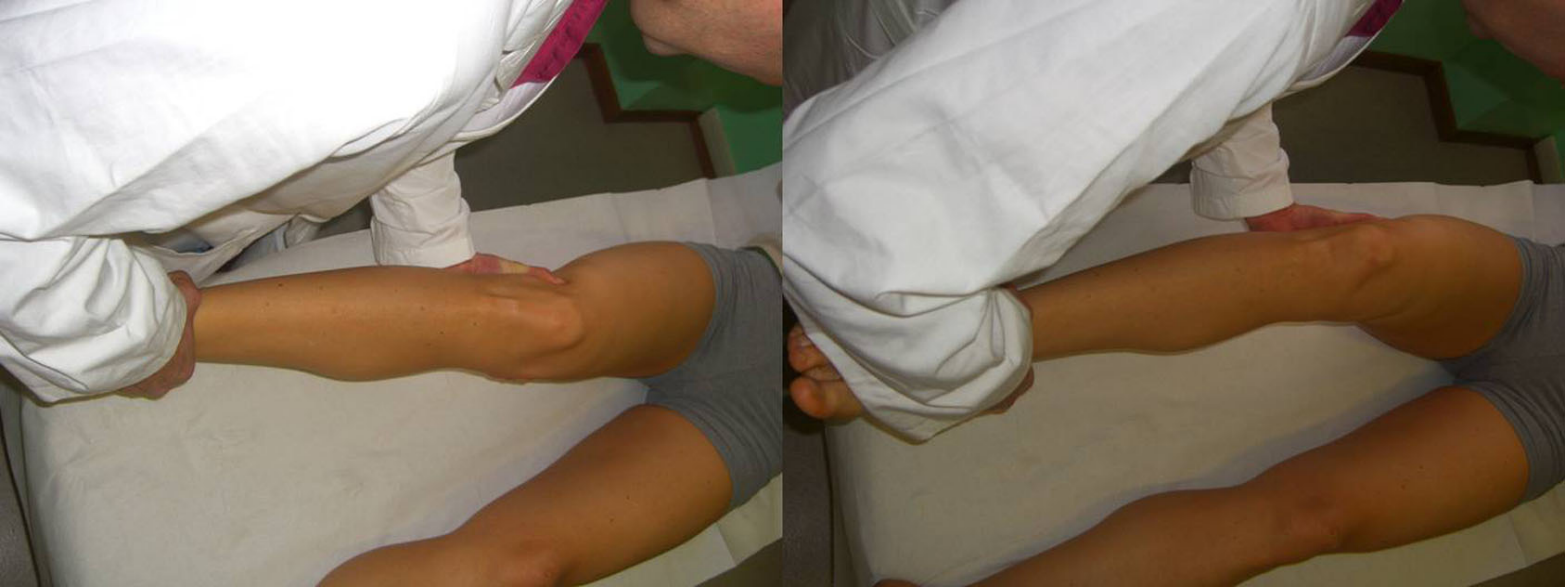
**Valgus and varus stress tests (Adapted with permission from: Rossi R, Bruzzone M, Dettoni F, Margheritini F: Clinical examination of the knee**. In: Orthopedic Sports Medicine, Principles and Practice. Edited by Margheritini F, Rossi R. Milan: Springer; 2010)

*Valgus (Abduction) stress test *and *Varus (Adduction) stress test *are among the most known and used knee tests. The key point in performing these tests is taking care not to perform them carelessly. The test should be carried out at 30° flexion rather than in full knee extension: by flexing the knee, all tendinous structures and posterior capsule are released allowing to test the MCL and LCL isolated. Palpating the joint line with one finger can be useful to determine the amount of opening. According to the American Medical Association (AMA), the amount of opening is graded as: grade I = 0 to 5 mm opening, with a hard endpoint; grade II = 5 to 10 mm, with a hard endpoint; grade III = over 10 mm opening, with a soft endpoint. Positivity of the test should not be referred to pain but to the degree of joint opening; in fact pain can be suggestive for partial rupture of the MCL, while a completely ruptured ligament is not stressed by the test therefore only mild pain is evoked (Figure 6).

*Cabot's manoeuvre *is another stress test, that evaluates the LCL. The knee is held in a 'figure of four' position, while giving a varus stress to the joint: the LCL, when intact, can be distinctively palpated as a tight chord stretched between the fibular head and the lateral epicondyle.

While keeping the patient's knee in this position the figure of four meniscal stress manoeuvre can also be perfomed (for details, see previously: figure of four meniscal stress manoeuvre).

**Figure 7:**
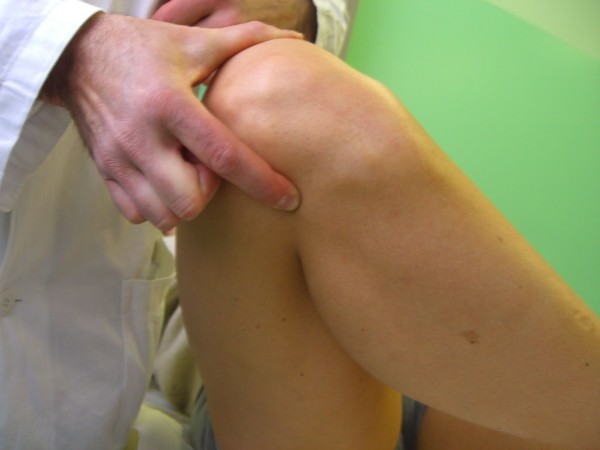
**Cabot's manoeuvre (Adapted with permission from: Rossi R, Bruzzone M, Dettoni F, Margheritini F: Clinical examination of the knee**. In: Orthopedic Sports Medicine, Principles and Practice. Edited by Margheritini F, Rossi R. Milan: Springer; 2010).

Cabot's manoeuvre and figure of four manoeuvre can raise severe pain, and are difficult to perform in an acute setting (Figure 7).

#### Slide Tests

With these tests the examiner slides the tibia, trying to subluxate it from the distal femur.

*Anterior and Posterior Drawer Test: *the most commonly used test for ACL and PCL evaluation, they are easy to perform, but require some attention to avoid mistakes and for correct interpretation. The tests have to be carried out in three different tibial rotational positions: neutral and at 30° of internal and external rotation. Internal rotation tightens the PCL and the posterolateral corner, so that the anterior drawer can become negative in this position. Anterior and posterior drawer test are performed simultaneously, and the examiner has to take care to rule out the amount of anterior and posterior tibial translation. Indeed in some cases when a PCL deficient knee has a posteriorized starting position, the reduction to a neutral postion can mimic an anterior drawer test: careful evaluation is required to avoid this mistake. In order to determine the correct starting point, palpation can be useful: in the neutral position the tibial plateau and the medial condyle face one another, with a slight anterior step-off of the tibia (approximately 0.5 - 1 cm); this has to be taken as the "zero point" for anterior and posterior drawer evaluation.

In an acutely swollen knee the test can be done keeping the knee in a less flexed position, at 60 to 80° thus avoiding excessive pain due to haemarthrosis.

**Figure 8:**
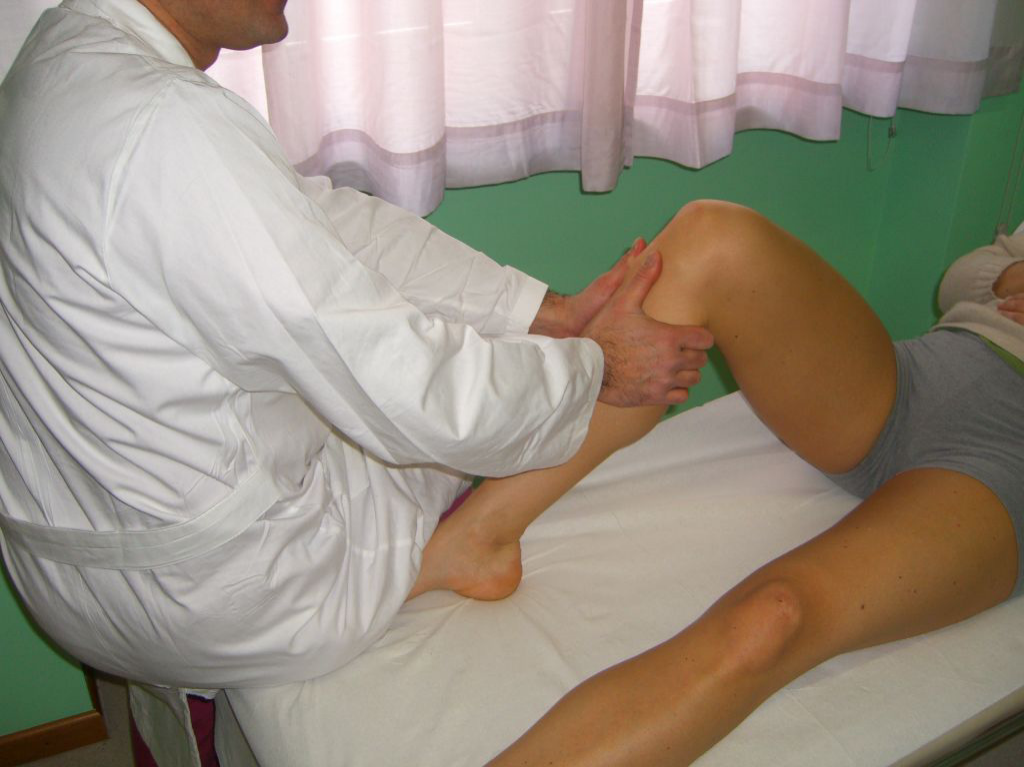
**Anterior Drawer test (Adapted with permission from: Rossi R, Bruzzone M, Dettoni F, Margheritini F: Clinical examination of the knee**. In: Orthopedic Sports Medicine, Principles and Practice. Edited by Margheritini F, Rossi R. Milan: Springer; 2010).

The menisci can mimic a hard stop, giving a false negativity to the test, when they engage in the joint space under the femoral condyles during the anterior dislocation movement. This 'doorstop' effect is more often given by the lateral meniscus, rather than the medial meniscus (Figure 8).

**Figure 9:**
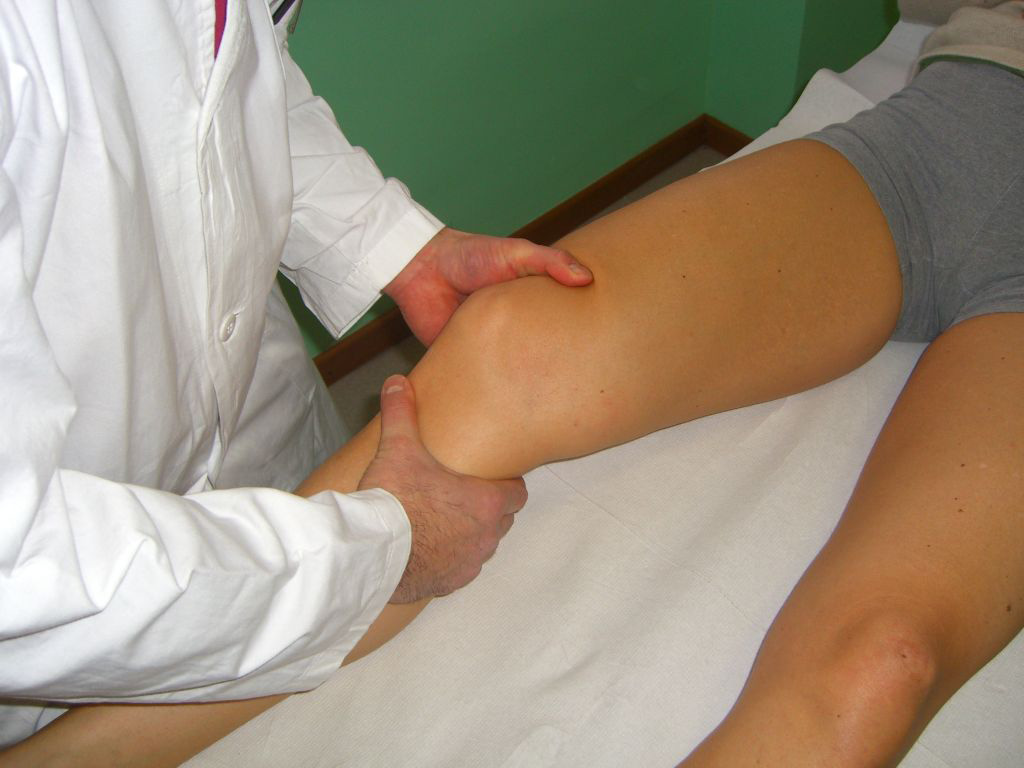
**Lachman Test (Adapted with permission from: Rossi R, Bruzzone M, Dettoni F, Margheritini F: Clinical examination of the knee**. In: Orthopedic Sports Medicine, Principles and Practice. Edited by Margheritini F, Rossi R. Milan: Springer; 2010).

The *Lachman test *is the test for ACL evaluation easier to be performed in all settings: it can be particularly useful in those cases when the knee is examined in the first days after injury, with the knee swollen and highly painful. The test is performed holding the knee in full extension and at 30° flexion, and slightly externally rotated. As in the drawer test, besides the amount of anterior dislocation it is important the quality of the endpoint: a soft stop is highly predictive for ACL rupture, while a hard stop can indicate an intact ACL, even in case of a sensible amount of tibial traslation (Figure 9).

The use of mechanical quantification of the tibial translation with measure instruments such as the KT-1000 (R) is useful for follow-up but not for diagnostic purposes.

The posterior Lachman test evaluates the PCL, with less efficacy than anterior Lachman.

**Figure 10:**
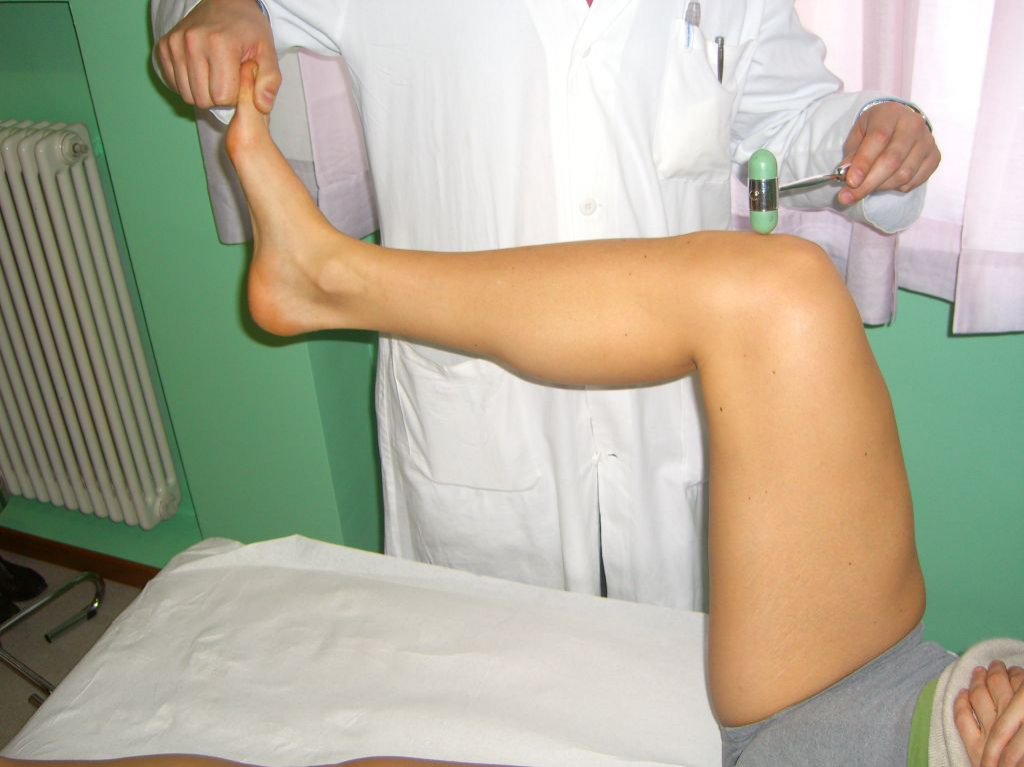
**Tibia step-back test for PCL (Adapted with permission from: Rossi R, Bruzzone M, Dettoni F, Margheritini F: Clinical examination of the knee**. In: Orthopedic Sports Medicine, Principles and Practice. Edited by Margheritini F, Rossi R. Milan: Springer; 2010)

Many test evaluate the tibial "sag", or subluxation, that can be encountered in PCL deficient knees: with the knee flexed, the tibia falls in a posterior subluxated potion; by contraction of the extensor apparatus this subluxation is reduced (anteriorly). The tibia can be held in three positions: with the patient supine, hip flexed at 90° and knee flexed at 90° (Figure 10); in the drawer position; with the knee slightly flexed as in Lachman's test. In these positions, a tibial draw back is noted (particularly in the first position: *Passive Tibial Sag sign*). Then the tibia is actively reduced by contraction the quadriceps muscle.

**Figure 11:**
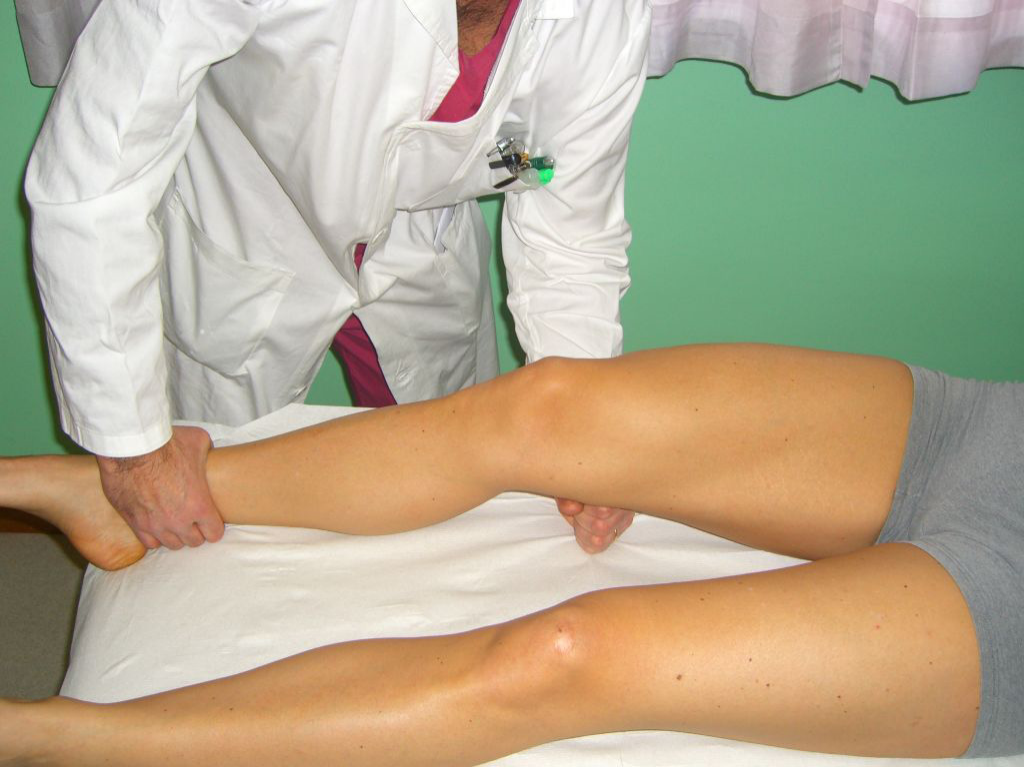
**Quadriceps Active Test (Adapted with permission from: Rossi R, Bruzzone M, Dettoni F, Margheritini F: Clinical examination of the knee**. In: Orthopedic Sports Medicine, Principles and Practice. Edited by Margheritini F, Rossi R. Milan: Springer; 2010).

In *Quadriceps Active Test *the patient is asked to contract the muscle maintaining the knee in a flexed position: this pulls upward the tibia, obliterating the sag (Figure 11).

In the Lachman-type position, the patient is asked to lift his leg against resistance (*Active Resisted Extension Test*).

In the drawer-type position, the patient is asked to lift his leg against resistance (*Active Resisted Extension Test II*), or contraction of quadriceps muscle is obtained by evoking the patellar reflex (*Patellar Reflex Reduction Test*).

#### Pivot Shift (jerk) Tests

These tests evaluate the rotatory instability that affects ACL deficient patients: this determines discomfort or frank pain with a shift or jerk of the knee joint, usually felt when squatting or changing direction.

An isolated ACL rupture produces a slight shift, often highly uncomfortable for the patients, while a posterolateral corner lesion is required to determine a huge, visible and sometimes audible jerk.

These tests are painful, and most of the times after the first attempt the test is no more reproducible, but they are the most effective in detecting an ACL rupture. In the hours before the injury, as the knee starts to swell, the tests becomes more and more difficult to be performed, and painful, so it has to be carried out in the most acute setting or in the chronic one.

McIntosh firstly described the test as the 'pivot shift' test *(McIntosh's Pivot Shift (Jerk) Test)*, quoting a hockey player with an unstable ACL deficient knee who reported: -when I pivot, my knee shifts-.

**Figure 12:**
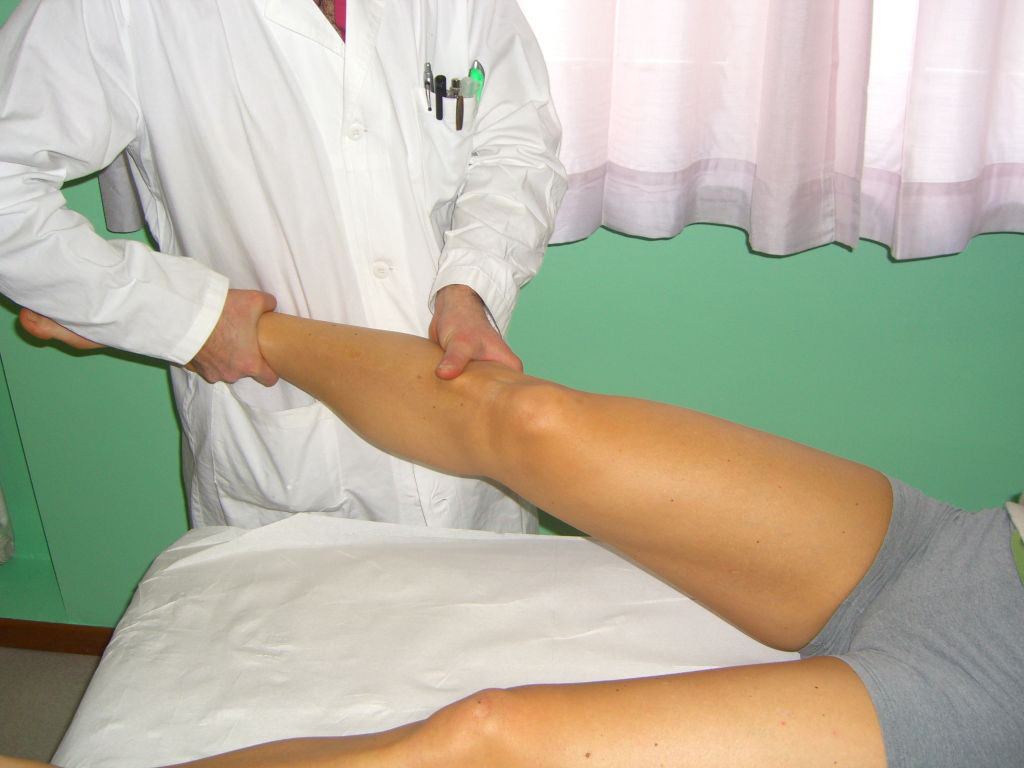
**Pivot Shift (Jerk) Test (Adapted with permission from: Rossi R, Bruzzone M, Dettoni F, Margheritini F: Clinical examination of the knee**. In: Orthopedic Sports Medicine, Principles and Practice. Edited by Margheritini F, Rossi R. Milan: Springer; 2010)

The mechanism of these tests is that in the first degrees of flexion the tibial plateau forced in a valgus and internal rotation stress subluxes anteriorly, then at about 30° flexion, it suddenly reduces posteriorly as the iliotibial band passes posterior to the center of rotation pushing backwards the tibial plateau inside the joint line (Figure 12).

In *Noyes' Glide Pivot Shift Test *the tibial subluxation is achieved not by internally rotating the leg, but rather by compressing the tibia axially towards the femur and lifting it anteriorly. The examiner tries to dislocate the whole tibial plateau (antero-posterior instability), not only the lateral aspect (rotatory instability), so a 'glide', rather than a clear clunk is evoked.

*Hughston's Jerk Test *produces the subluxation by extending the knee from the flexed position, applying the same valgus and internal rotation stress as in Noyes' test.

**Figure 13:**
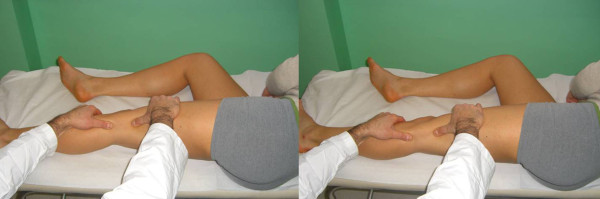
**Slocum's Anterolateral Rotary Instability (ALRI) Test (Adapted with permission from: Rossi R, Bruzzone M, Dettoni F, Margheritini F: Clinical examination of the knee**. In: Orthopedic Sports Medicine, Principles and Practice. Edited by Margheritini F, Rossi R. Milan: Springer; 2010)

The *Slocum's Anterolateral Rotary Instability (ALRI) Test *is performed with the patient on the side, in a semi-lateral position, resting on the unaffected limb, with the affected knee extended and the limb supported by only the heel resting on the examining table. In this position the foot and tibia rotate internally, translating anteriorly the lateral tibial plateau. Vertical (valgus) stress is applied to the knee, then the knee is progressively flexed. In the first 20 degrees of flexion the tibia subluxes, while at approximately 40° it reduces, with a sudden reduction shift (or clunk); a finger placed at the joint line can help detecting the reduction. The position of the pelvis, held on the side and slightly posteriorly, avoids the rotational bias of the hip. This test is reported to be more effective than other pivot shift tests, and less painful for the patient (Figure 13).

The *Reverse Pivot Shift Sign *evokes the same shift as in pivot shift signs, but for PCL deficient knees: in these cases the lateral tibial plateau subluxes posteriorly when the tibia is stressed in external rotation and valgus, and reduces in extension.

The test can also be performed in the reverse direction, from the extended reduced position to the flexed subluxed one.

#### External Rotation Tests

These tests evaluate the posterolateral corner: a PLC deficient knee presents an external rotatory instability. PLC lesions are often associated to ACL or PCL tears, so it not uncommon to underestimate or misdiagnose a PLC lesion; the following tests are intended to electively evaluate the posterolateral corner [13,14].

**Figure 14:**
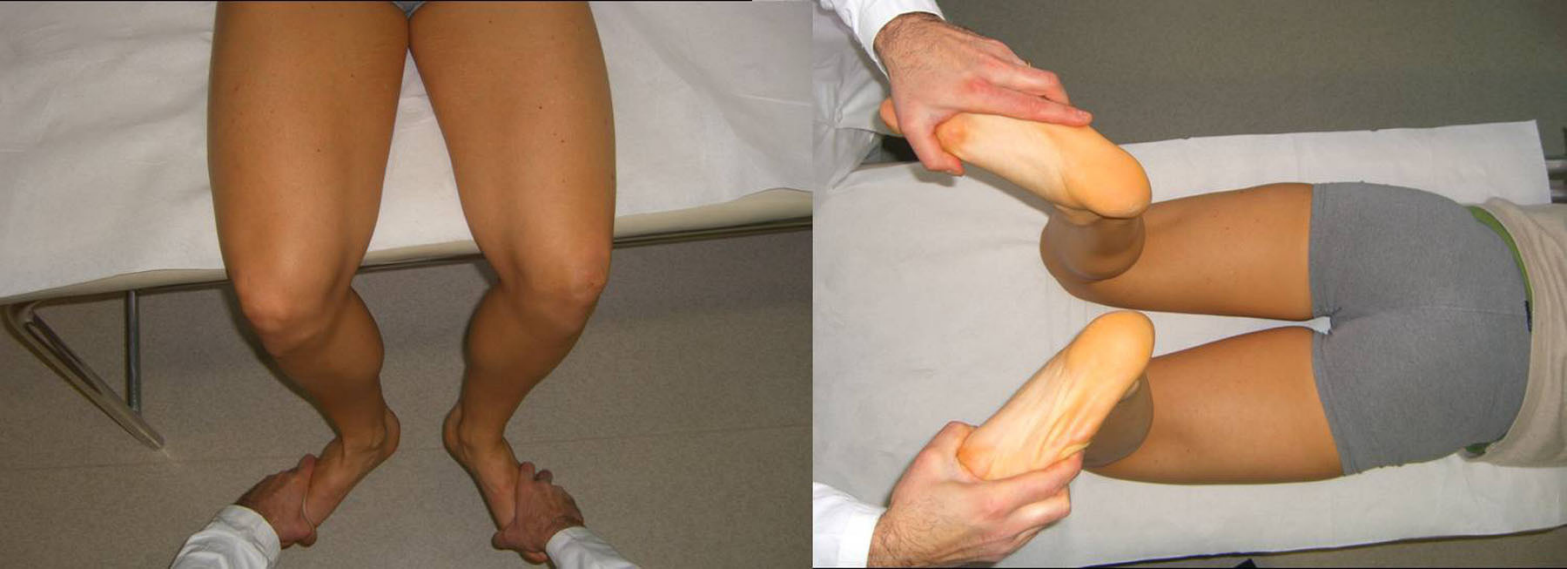
**Tibial External Rotation (Dial) Test (Adapted with permission from: Rossi R, Bruzzone M, Dettoni F, Margheritini F: Clinical examination of the knee**. In: Orthopedic Sports Medicine, Principles and Practice. Edited by Margheritini F, Rossi R. Milan: Springer; 2010).

The *Tibial External Rotation (Dial) Test *evaluates the amount of increased passive external rotation of the tibia in different positions of the knee. The supine position is more comfortable for the patient, but in the prone position the hip is held in its position by the patient's weight, thus eliminating the rotator effect of the hip. The leg should be held at 30° and 90° flexion, while in full extension the lateral gastrocnemius tendons are tightened, and reduce the external rotation drive. The test is positive when the affected knee rotates externally 10° more than the unaffected knee. In the flexed position the proximal tibial not only rotates externally, but subluxes posteriorly as well: reducing the tibia on the lateral femoral condyle with a finger further increases the amount of external rotation (Figure 14).

**Figure 15:**
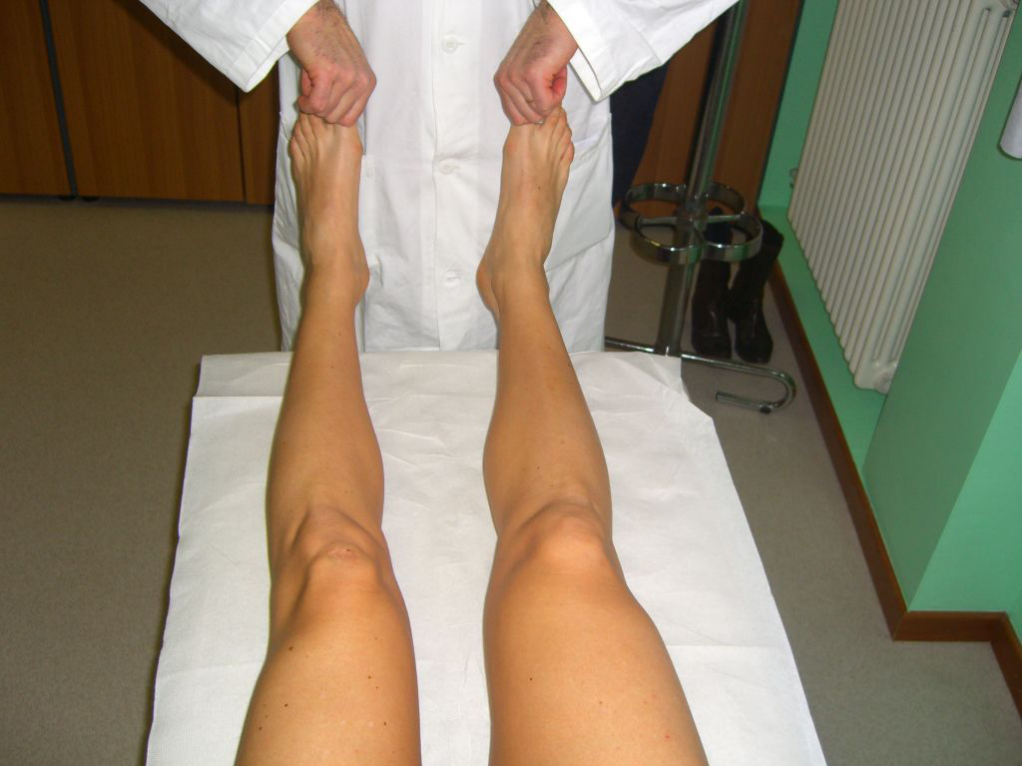
**External Rotation Recurvatum Test (Adapted with permission from: Rossi R, Bruzzone M, Dettoni F, Margheritini F: Clinical examination of the knee**. In: Orthopedic Sports Medicine, Principles and Practice. Edited by Margheritini F, Rossi R. Milan: Springer; 2010).

The *External Rotation Recurvatum Test *evaluates the PLC and the posterior capsule. The knee is progressively extended from 10° flexion to the maximum extension, while rotating externally. Positivity is given by the combination of increased external rotation and hyperextension (recurvatum). Alternatively, the test can be evaluated by lifting the extended lower limbs by the toes, thus applying a force combining varus, external rotation and recurvatum (Figure 15).

A great amount of recurvatum suggest an associated PCL rupture.

The *Posterolateral External Rotation (Drawer) Test *is a combination of the posterior drawer and external rotation tests: with the knee flexed at 30° and then at 90°, the tibia is forced posteriorly and in external rotation subluxating the tibia. If subluxation occurs at 30° but not at 90° an isolated PLC injury is supposed, while if subluxation occurs also at 90° a combined PCL and PLC is suspected.

#### Injury Patterns

Different injury patterns show different tests positivity. The patterns and relative tests are listed in the table below, in order of sensitivity (see table 1, below) [15].

**Table 1:** Injury patterns, and relative tests.

Injury Pattern	Injured Structure	Tests
Anteromedial Instability	ACL + MCL + MM	valgus stress, anterior drawer, Lachman
Anterolateral Instability	ACL + LC + LM	valgus stress, anterior drawer, Lachman, pivot shift
Posterolateral Instability	PLC	external rotation, dial, recurvatum, posterolateral drawer
Posteromedial Instability	MCL + ACL + PMC	valgus stress, posterior drawer, Lachman
Anteromedial + Anterolateral Instability	ACL + MCL + lateral capsule (PCL intact)	anterior drawer, Lachman, pivot shift, valgus stress
Posteromedial + Posterolateral Instability	ACL + MCL +PLC (PCL intact)	anterior drawer, Lachman, pivot shift, valgus stress, varus stress

Tests are reported in order of sensibility (Adapted with permission from: Rossi R, Bruzzone M, Dettoni F, Margheritini F: Clinical examination of the knee. In: Orthopedic Sports Medicine, Principles and Practice. Edited by Margheritini F, Rossi R. Milan: Springer; 2010).

ACL: Anterior Cruciate Ligament; MCL: Medial Collateral Ligament; MM: Medial Meniscus; LC: Lateral Capsule; LM: Lateral Meniscus; PLC PosteroLateral Corner; PMC: PosteroMedial Corner; PCL Posterior Cruciate Ligament

## Conclusions

This paper reviews the most known and used tests and signs for knee examination, outlining the importance of performing the tests in the correct way, in the right setting and timing and giving the correct interpretation. Knowing well these tests, the surgeon has a powerful tool for diagnosis and followup of the pathologies involving patellofemoral compartment, meniscal and chondral lesions and instability of the knee.

## Competing interests

The authors declare that they have no competing interests.

## Authors' contributions

All authors made substantive intellectual contributions to a this paper. All authors read and approved the final manuscript.
